# A New Case of Spontaneous Regression of Inflammatory Hepatic Pseudotumor

**DOI:** 10.1155/2011/139125

**Published:** 2011-03-22

**Authors:** Hichem Jerraya, Slim Jarboui, Hassen Daghmoura, Abdeljelil Zaouche

**Affiliations:** Charles Nicolle's Hospital, Surgical Unit A, Tunis, Tunisia

## Abstract

*Introduction*. Inflammatory pseudo-tumors (IPT) of the liver are rare and difficult to diagnose, because mimicking malignant tumors. *Aim*. We report a case of IPT of the liver wich diagnosis was made on clinical, radiological and evolutif features. *Observation*. A 15-year-old man had a 4-month history of abdominal pain in the right upper quadrant with fever and cought. Two successives ultrasonographies revealed a hypoechoic lesion occuping the segment VIII with 8 cm of diametre. Physical examination was normal. Laboratory investigation showed normal blood counts, liver function test and tumoral markers. Another ultrasonography was interpretated as normal. Tomodensitometry had showon a 3-cm lesion wich enhanced later after contrast injection. A second tomodensitometry done one mounth later described a 2-cm sub capsular heaptic lesion. *Discussion*. On routine activiy, pre operative diagnosis of IPT of the liver is difficut, and rarely made with certitude because mimicking a malignant tumor. In our cae report here, the analysis of previous history, of clinical, biological and radiological presentation, had permittes us to pose the diagnosis of PTI of the liver and this despite the absence of histological confirmation by percutaneous biopsy.

## 1. Introduction

The inflammatory pseudotumors of the liver (IPLs) are fibroblastic proliferations more or less fibrous, infiltrated by polymorphic inflammatory cells [1]. They are not so rare since the literature showed many edifying examples [2]. Their better characterization with the morphological examinations had permitted us these last years to make the diagnosis more often. This stresses, especially in face of the great progress realized in the field of surgery of malignant hepatic tumors, the great importance to pay attention to IPLs which the evolution is benign and does not necessitate any intervention. Thus, it is crucial to put on the right diagnosis and prevent unnecessary hepatic resection.

Hereby we present another case of spontaneous regression of IPL in which we recall diagnostic and evolutionary modalities.

## 2. Case Report

**Figure 1:**
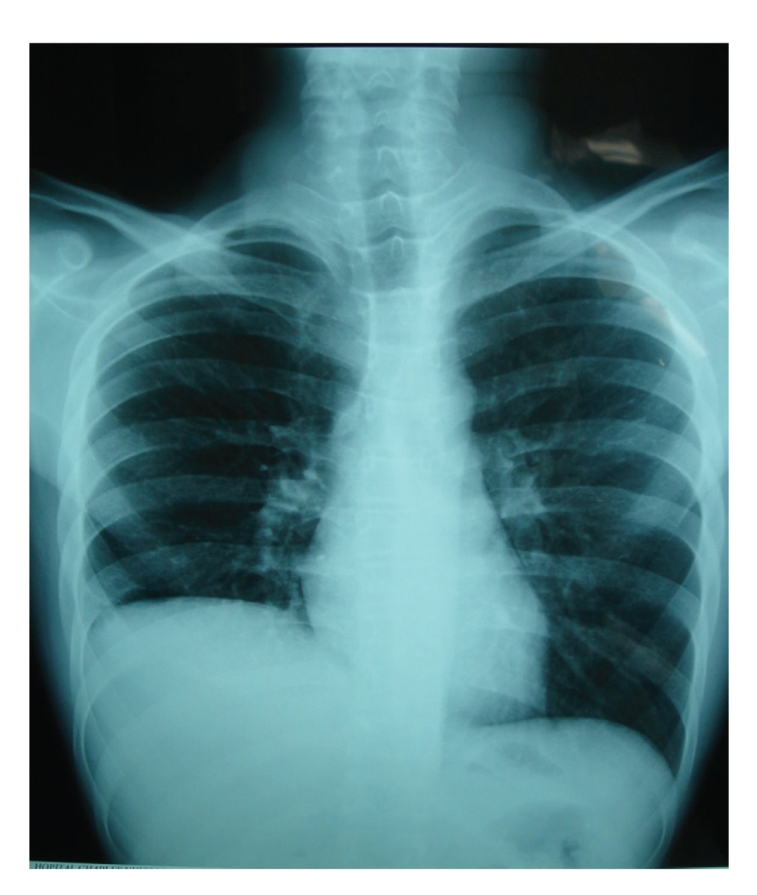
Elevation of the right diaphragmatic dome.

**Figure 2:**
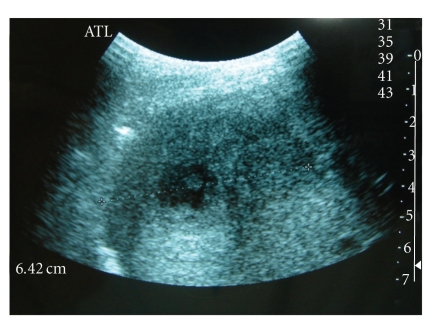
Hypoechoic and heterogeneous mass, localized in the hepatic dome.

We report a case of 15-year-old young man otherwise healthy who complains for 4 months of right upper quadrant pain accompanied with high fever and productive cough. Chest X-ray revealed an elevation of the right diaphragmatic dome (Figure 1). Abdominal ultrasonography showed a hypoechoic and heterogeneous mass, measuring 8 cm in diameter, localized in the hepatic dome (Figure 2). At his admission, the physical examination was normal. White blood cell count was 7500; c-reactive protein was normal too. Hepatic function test and tumor markers were within normal range (AFP and CEA).

**Figure 3:**
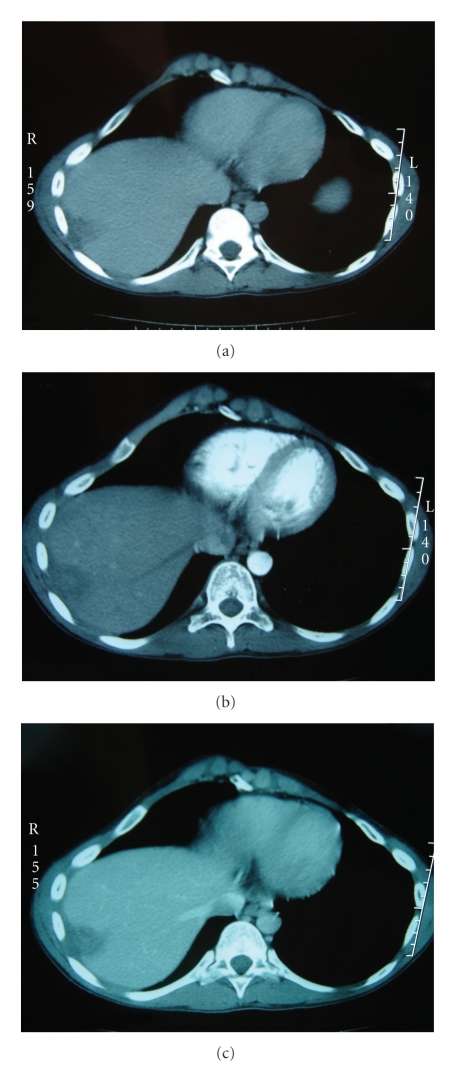
CT scan showing subcapsular lesion occupying segment VII and VIII and measuring 4 cm in diameter spontaneously low attenuated and having slight peripheral enhancement at delayed stage.

Abdominal tomography revealed a subcapsular lesion occupying segments VII and VIII and measuring 4 cm in diameter spontaneously hypodense and having slight peripheral enhancement at delayed stage (Figure 3).

**Figure 4:**
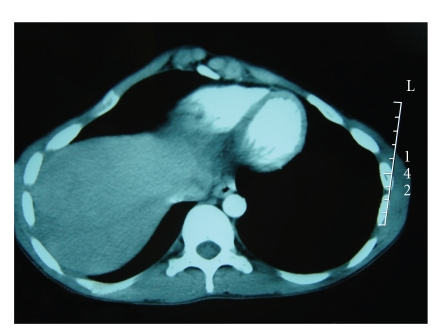
CT scan showing a decrease in tumor size which is evaluated to be 2 cm.

A second tomography done one month later described the same lesion with the same characteristics but with a dramatic decrease in size which is evaluated to be 2 cm in diameter (Figure 4). This spontaneous regression in size associated with radiological characteristics of a partially fibrous lesion permitted us to make the diagnosis of IPL and to abstain from any therapeutics. The patient is doing well and another radiologic control is foreseen in 6 months.

## 3. Discussion

The pathogenesis of IPL remains uncertain. It would be in relation with an exaggerated or inadequate inflammatory response of an existing microorganism in the portal circulation [3]. Abbey-Toby et al. [4] distinguished two clinicopathological entities which have different clinical and radiological forms: the first one consists of both fusiform cells and inflammatory polymorphic cells associated with adjacent fibrous portal endophlebitis and accompanied besides that by an inflammatory syndrome. In return, the second form which is encapsulated has abundant central necrosis and it is always clinically asymptomatic. It would be the result of a chronic inflammatory processes and this corresponds to the healing version of IPL. This evolutionary form explains the diminution in size lesion observed in our patient. This evoked several reported cases in literature where regression of IPL either spontaneously or by an anti-inflammatory or antibiotics [2, 5–11] is reported. 

Since the IPL are known as benign lesions [2] and that its natural history progresses towards regression, it is essential to make the exact diagnosis and to not consider it as a malignant tumor that may lead to unnecessary resection. However, diagnosis is not easy due to the absence of specific radiological signs and to the variability of the radiologic aspects which are related to the evolutionary stage of IPL [4]. In the current case, the main characteristic that helped to make the diagnosis is the regression of the tumor size on two successive CT scan. The diagnosis should be discussed also when a liver mass has the characteristics of a lesion with a fibrous component with late contrast or necrotic. Indeed, the IPL is hypoechoic on US and has well-defined borders. On noncontrast CT study, the lesion reveals low attenuation and a moderate enhancement following contrast administration in the periphery and in late stage. On MRI, IPL is frequently hypointense in T1-weighted images, hyperintense in T2-weighted images, and of heterogenic uptake after gadolinium injection [12]. However, these features are variable and the diagnosis is often evoked in the absence of signs for common benign and malignant liver lesions (Table 1).

In terms of radiologic analysis, the diagnosis of IPL should be evoked every time that we are faced with partially fibrous hepatic mass with late uptake of contrast material and in absence of signs for specific hepatic tumor [12]. In this stage the diagnosis may be put on by the presence of regression in size of the hepatic mass as it was the case for our patient and two other cases reported by Yamaguchi et al. [5]. In total, the combination of radiologic suggestive signs on CT scan with a regression of tumor size on successive morphological examinations eliminates all differential diagnoses and can retain the diagnosis of IPT. Otherwise, in the absence of one of these criteria, percutaneous needle biopsy of the tumor is indicated [11]. The pathologic diagnosis is based on the presence of densely hyalinized collagenous tissue with an inflammatory infiltrate of predominantly plasma cells. Some portions of the stroma show fibroblasts in interlacing laminated or whorled patterns. Typical changes are seen in large and medium-sized veins with inflammation involving the vessel walls and filling of the lumen with connective tissue containing capillaries and inflammatory cells. Many veins show complete obliteration of the lumen. In contrast, arteries show no major alterations [2]. 

It has not been established whether steroid administration accelerates resolution of IPL but it may reduce the systemic symptoms of the inflammatory syndrome [2]. Most often the tumor regresses spontaneously and only a radiological monitoring should be introduced until complete healing of the lesion.

## 4. Conclusion

The IPL are benign lesions which show spontaneous regression. The preoperative diagnosis is difficult since the radiological presentation is taken for a malignant tumor. Attention should always be paid in case of partially fibrous hepatic mass with late contrast uptake and without any specific sign in order to avoid any unnecessary surgery.

## Figures and Tables

**Table 1:** Imaging features of common hepatic lesions.

	US	CT scan	MRI
Hemangioma	(i) hyperechoic	(i) low attenuation on the noncontrast CT	hyperintense on T2
	(ii) well-defined or lobulated borders	(ii) peripheral nodular enhancement	

Focal nodular hyperplasia (FNH)	(i) isoechoic	early enhancement with characteristic central scar	hypointense with central scare which is hyperintense on T2
	(ii) well-defined borders		

Hepatic adenoma	discretely hypoechoic	early enhancement	hyperintense on T1 and T2

Hepatocellular carcinoma	(i) hypoechoic, heterogeneous	(i) early and heterogeneous enhancement	hypointense on T1 and hyperintense on T2
	(ii) portal thrombosis	(ii) hypodense, with only the capsule enhancing, on delayed-phase	
		(iii) portal vein occlusion from venous invasion with intraluminal tumor present and expansion of the vessel	

Metastatic disease	(i) multiple hypoechoic lesions	thick and irregular rim with enhancement	(i) mildly hyperintense to liver
	(ii) lesions with a hypoechoic rim or halo pattern		(ii) irregular or rim enhancement

IPT of the liver	(i) usually unique	(i) low attenuation on the noncontrast CT	(i) hypointense on T1, hyperintense on T2
	(ii) hypoechoic	(ii) late and peripheral enhancement	(ii) heterogenic enhancement
	(iii) well-defined borders	(iii) venous occlusion from gross thickening of the wall of the vein	
